# The rise of hikikomori research: a bibliometric analysis of an emerging global phenomenon

**DOI:** 10.3389/fpsyg.2026.1862953

**Published:** 2026-07-24

**Authors:** Fatih Cebeci

**Affiliations:** 1 Department of Social Work, Faculty of Health Sciences, Istanbul Medipol University, Istanbul, Türkiye; 2 UNEC Social Work and Social Innovations Research Center, Azerbaijan State University of Economics, Baku, Azerbaijan

**Keywords:** bibliometric analysis, hikikomori, mental health, research trends, science mapping, social withdrawal

## Abstract

**Introduction:**

Hikikomori, characterized by prolonged and severe social withdrawal, has attracted growing international attention as research has extended beyond its original Japanese context. This study examines the growth dynamics, citation impact, productivity patterns, collaboration structure, and conceptual and intellectual development of the hikikomori literature.

**Methods:**

Data have been retrieved from the Web of Science Core Collection and Scopus databases. Following screening and deduplication, 348 unique publications from 2002 to 2025 have been included. Performance analysis and science mapping techniques have been applied using the bibliometrix R package, Biblioshiny, and VOSviewer.

**Results:**

Publication output has remained limited during the early years, has become more visible after 2010, and has increased markedly after 2018, with the highest annual output recorded in 2025 (*n* = 62). Highly cited publications have included both core hikikomori studies and research related to internet addiction, digital behaviors, youth social withdrawal, and measurement. Scientific production has been concentrated among a limited group of authors and institutions, while 14 journals have formed the Bradford core. Japan has remained the dominant contributor, although substantial research activity has also been recorded in Italy, China, the United States, and other regions. International collaboration has been unevenly distributed and has remained less prominent than nationally based research production. Keyword analyses have revealed strong connections among hikikomori, social withdrawal, social isolation, depression, loneliness, mental health, and anxiety. Bibliographic coupling has revealed substantial overlap in shared reference bases, while thematic evolution has shown continuity around hikikomori and social isolation together with the later prominence of mental health, COVID-19, depression, and adolescent-related themes.

**Conclusion:**

The findings have indicated that hikikomori research has developed into a broader and increasingly international field in which clinical, psychosocial, developmental, cultural, and digital perspectives have been represented. Greater interdisciplinary and cross-cultural engagement has therefore been identified as an important direction for future research.

## Introduction

Hikikomori is a severe and prolonged form of social withdrawal characterized by marked social isolation in the home for at least 6 months and associated with significant functional impairment or distress (Kato et al., 2020). Although related forms of social withdrawal had been described earlier, the concept of hikikomori gained broad recognition in Japan following Saito (1998) work and was initially discussed largely within a Japanese sociocultural framework (Saito, 1998; Furlong, 2008; Teo, 2010; Teo and Gaw, 2010). Subsequent literature, including case reports, clinical studies, survey-based investigations, and review articles, has described a range of hikikomori-related phenomena, including clinically assessed cases, hikikomori-like presentations, and survey-identified patterns of severe social withdrawal, across diverse cultural contexts such as South Korea, China, Italy, Spain, France, Turkey, and the United States (Benarous et al., 2022; Dong et al., 2022; Hu et al., 2022; Kasak et al., 2022; Kato et al., 2012; Malagón-Amor et al., 2018; Orsolini et al., 2023). This growing international evidence has contributed to a shift from viewing hikikomori primarily as a Japan-specific phenomenon toward recognizing its broader cross-cultural relevance and potential global mental health implications (Dong et al., 2022; Kato et al., 2012; Kato et al., 2020). The estimated lifetime prevalence in Japan ranges between approximately 1.2 and 2%. Studies based on general population samples indicate that hikikomori-like social withdrawal is not limited to Japan but is also observed in different countries (Nonaka et al., 2022; Pozza et al., 2019; Teo and Gaw, 2010).

Hikikomori is a multidimensional phenomenon associated with substantial psychological, social, and functional difficulties, extending beyond individual distress to broader disruptions in education, employment, and social participation (Kato et al., 2024; Muris and Ollendick, 2023). Prolonged social withdrawal can involve disengagement from education or employment, reduced social participation, and broader functional impairment (Kato et al., 2024; Muris and Ollendick, 2023; Tolomei et al., 2023). It has also been associated with depression, anxiety, suicidal ideation, and psychotic phenomena, particularly delusional experiences (Fong and Yip, 2023; Tolomei et al., 2023; Yasuma et al., 2021). Recent evidence has further linked higher hikikomori tendencies to lower perceived social support and psychological resilience, as well as to greater unemployment anxiety and learned meaninglessness. These findings point to the potential relevance of social resources, adaptive psychological capacities, unemployment-related concerns, and meaning-related processes in social withdrawal (Arslankoç et al., 2026; Cebeci et al., 2026). Adolescence and early adulthood represent particularly vulnerable developmental periods, during which identity formation and transitions into adult social and occupational roles are central (Hihara et al., 2022; Muris and Ollendick, 2023). Evidence linking identity distress to more severe trajectories of hikikomori symptoms suggests that social withdrawal during these periods may complicate participation in education, employment, and broader social life (Hihara et al., 2022; Muris and Ollendick, 2023). The COVID-19 pandemic has further intensified concerns about prolonged social withdrawal. Lockdowns, reduced in-person contact, and greater reliance on online lifestyles have been discussed as conditions that may increase the risk of hikikomori-like withdrawal, which has also been considered a potential longer-term mental health consequence of the pandemic (Herold et al., 2021; Kato et al., 2024). Research on hikikomori has increased markedly over the past decade, with a growing body of literature addressing its definition, prevalence, and associated factors. Kato et al. (2020) proposed an internationally applicable diagnostic framework, while Teo et al. (2023) developed a structured assessment tool to support this framework. Reviews by Nonaka et al. (2022, 2025) have deepened the understanding of the demographic and clinical characteristics of hikikomori, and Pupi et al. (2025) have provided a detailed examination of its psychiatric comorbidities. In addition, Tateno et al. (2019) and Lin et al. (2025) have highlighted its associations with technology use and digital addiction, whereas Santona et al. (2023) have emphasized psychosocial dimensions such as attachment and environmental sensitivity. This body of work indicates that hikikomori research has not only expanded in volume but has also become more diverse in its theoretical and methodological approaches, moving toward a more integrated understanding of the phenomenon.

This growing interest has also led to a limited number of studies that examine the hikikomori literature using bibliometric approaches. Cai et al. (2023) analyzed 297 publications retrieved from the Web of Science database, identifying publication trends, leading authors and countries, and keyword networks. Similarly, Hussain (2023) examined 286 publications based solely on the Scopus database, focusing on the developmental trajectory and academic distribution of the field. Neoh et al. (2023), using CiteSpace, conducted a scientometric analysis of 302 publications, mapping document co-citation clusters and country collaboration networks. While these studies provide valuable insights into the structural characteristics of the hikikomori literature, they present certain limitations in terms of database coverage and analytical depth. Relying on a single database increases the risk of database-specific bias, and the restriction of their data to publications up to 2022 limits their ability to capture the rapid growth observed in recent years. In addition, comprehensive analyses that integrate author productivity, source distribution, and the intellectual structure of the field through approaches such as bibliographic coupling remain largely absent. These limitations make it difficult to develop a holistic understanding of the field’s overall trends, leading contributors, core publication sources, and the evolution of its research agenda.

### The present study

Bibliometric methods are well suited to addressing these gaps because they allow large bodies of literature to be examined systematically while revealing patterns of productivity, collaboration, intellectual relatedness, and thematic development (Donthu et al., 2021; Zupic and Čater, 2015). Accordingly, a comprehensive bibliometric analysis of publications retrieved from the Web of Science Core Collection and Scopus databases has been conducted to address the identified gaps. By integrating performance analysis with science mapping techniques, the growth dynamics, productivity patterns, source distribution, collaboration structure, and conceptual and intellectual development of the hikikomori literature have been examined. The study has been guided by the following research questions:

- RQ1. How has the hikikomori literature evolved over time in terms of annual publication output and citation patterns across publication years?
- RQ2. Which publications have had the greatest citation impact in the hikikomori literature, and what research areas are represented among the most highly cited studies?
- RQ3. Who are the most productive and influential authors in the hikikomori literature, and which institutions show the highest levels of research productivity?
- RQ4. How are publications distributed across journals, and which sources constitute the core of the field based on Bradford’s Law?
- RQ5. How is hikikomori research distributed geographically, and what patterns of collaboration are observed at the author and country levels?
- RQ6. What conceptual and intellectual structure emerges from keyword co-occurrence, bibliographic coupling, and thematic evolution analyses?

## Methods

### Research design

This study was designed as a descriptive and exploratory bibliometric analysis to examine the development, collaboration patterns, and conceptual structure of the hikikomori literature. Bibliometric methods enable the systematic and quantitative analysis of large bodies of scientific publications and are widely used to identify intellectual structures, research trends, and thematic evolution within a field (Donthu et al., 2021; Zupic and Čater, 2015). Bibliometric analysis also allows for the examination of publication trends, citation patterns, and collaboration networks, providing a comprehensive understanding of how knowledge evolves within a research field (Sangadji, 2023). By integrating performance analysis with science mapping techniques, the study provides a structured and reproducible overview of the hikikomori research domain. Performance analysis focuses on the productivity and impact of research actors, while science mapping explores the relationships among them and reveals the intellectual structure of the field (Moral-Muñoz et al., 2020). The analysis was conducted using the bibliometrix R package (via Biblioshiny) and VOSviewer, which are widely used tools that combine analytical capabilities with network visualization to map bibliometric relationships (Du et al., 2024; Moral-Muñoz et al., 2020).

### Data sources

The bibliometric dataset was compiled from two major international indexing databases: the Web of Science Core Collection and Scopus. These databases were selected because of their extensive literature coverage and complementary indexing structures. The use of multiple databases was considered appropriate for improving literature coverage and reducing database-specific bias (Zupic and Čater, 2015). All records were exported in BibTeX format to facilitate data integration, cleaning, and subsequent analyses. The searches were conducted on 15 March 2026. To avoid the inclusion of an incomplete publication year, records published in 2026 were excluded, and the final analytical period was therefore limited to 2025 and earlier years.

### Search strategy

#### Web of science

In the Web of Science Core Collection, the search was conducted in the Topic field (TS), which includes title, abstract, author keywords, and Keywords Plus. The search query was structured as follows:

TS = (“hikikomori” OR “severe social withdrawal” OR “prolonged social withdrawal” OR “extreme social withdrawal” OR “pathological social withdrawal” OR “social withdrawal syndrome”).

Results were limited to English-language publications and document types of article and review, ensuring the inclusion of peer-reviewed journal studies only.

#### Scopus

In Scopus, the search was conducted in the Title, Abstract, and Keywords fields using the following query:

TITLE-ABS-KEY (“hikikomori” OR “severe social withdrawal” OR “prolonged social withdrawal” OR “extreme social withdrawal” OR “pathological social withdrawal” OR “social withdrawal syndrome”).

Filters were applied for document type (article and review), source type (journal), and English language.

### Study selection and data preparation

**Figure 1:**
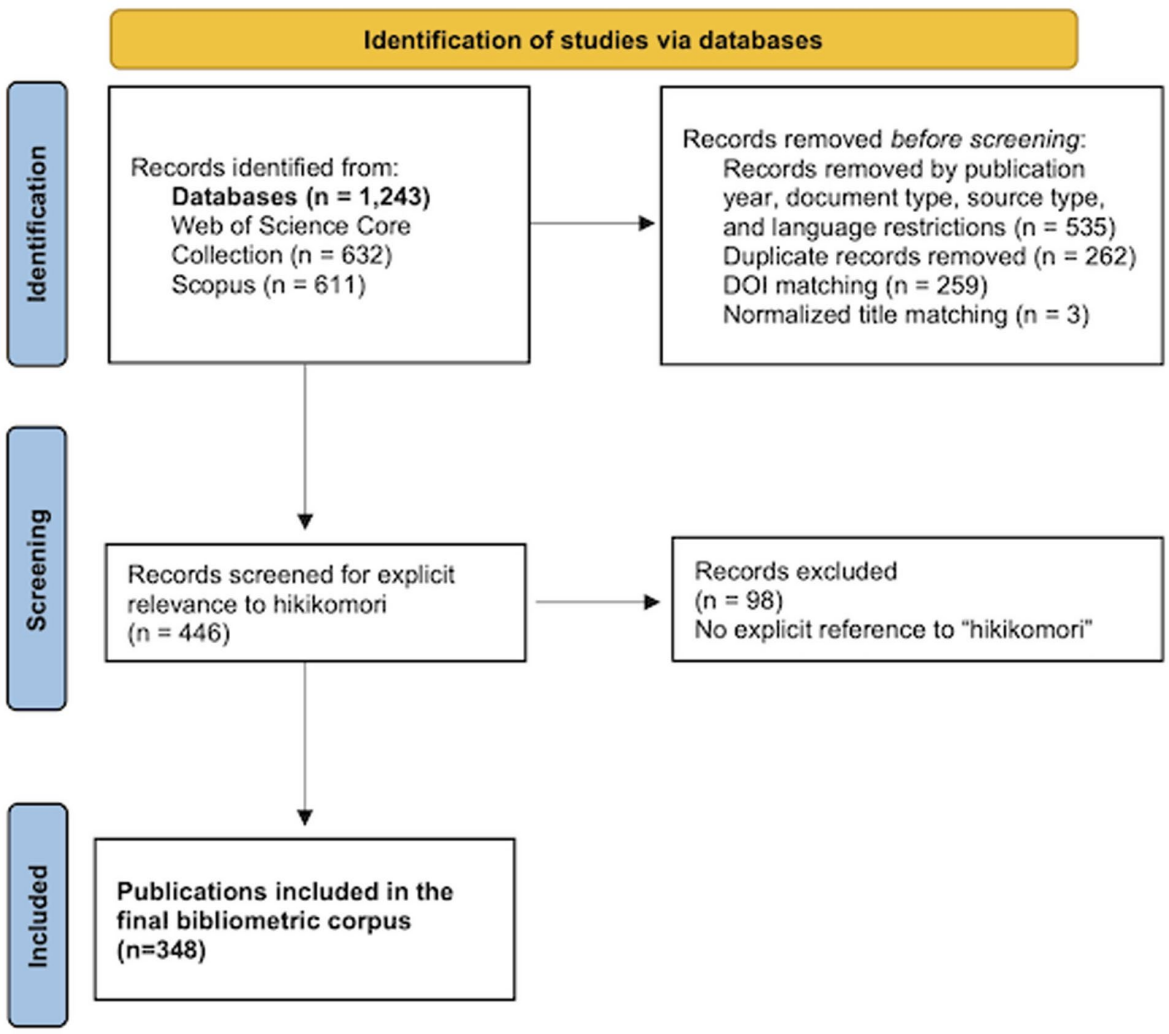
PRISMA-style flow diagram of the bibliometric data collection process. Adapted from the PRISMA 2020 flow diagram (Page et al., 2021), under the CC BY 4.0 license. Source: https://www.prisma-statement.org/prisma-2020-flow-diagram.

The database searches initially yielded 1,243 records, comprising 632 records from Web of Science and 611 from Scopus. No lower year limit was applied. However, records published in 2026 were excluded to avoid the inclusion of an incomplete publication year. After document type, source type, and language restrictions were applied, 708 records were retained, including 341 from Web of Science and 367 from Scopus. The two datasets were then merged, and duplicate records were removed in two stages. First, 259 duplicate records were identified and removed through DOI-based matching. Records with missing or incomplete DOI information were subsequently examined using normalized title comparisons, resulting in the identification and removal of three additional duplicates. Following deduplication, 446 unique records remained for eligibility assessment. The remaining records were then screened for explicit relevance to hikikomori. The initial search strategy included “hikikomori” together with broader social withdrawal terms. During eligibility screening, however, the analytical scope was restricted to publications that explicitly referenced “hikikomori” in the title, abstract, or author keywords. This criterion was used to delimit the corpus to literature directly engaging with the hikikomori concept. Records using only broader social withdrawal terminology without an explicit reference to hikikomori were excluded. A total of 98 records were excluded at this stage, resulting in a final bibliometric corpus of 348 unique publications published between 2002 and 2025. The study selection process was documented using a PRISMA-style flow diagram adapted to the bibliometric context and is presented in Figure 1.

### Keyword cleaning and standardization

Author keywords were used in the keyword co-occurrence analysis because they were considered to reflect the conceptual focus of the publications more directly. Indexed keywords were retained during data preparation to assist with terminology checks and standardization, whereas the main co-occurrence network was constructed from author keywords to limit the influence of broad or database-generated terms. Before the network analysis was conducted, the keyword data were reviewed and standardized. Generic indexing terms and labels with limited conceptual relevance were removed where necessary. Obvious spelling inconsistencies and lexical variants were corrected, and singular and plural forms were harmonized when they referred to the same concept. Equivalent expressions were consolidated using a VOSviewer thesaurus file, while conceptually distinct terms were retained separately. For example, “depressive symptoms” was grouped under “depression,” and COVID-related variants were standardized as “COVID-19.” The term “hikikomori” was retained separately because of its specific conceptual position within the field. The cleaned set of author keywords was then analyzed in VOSviewer using the full counting method, with a minimum occurrence threshold of four.

### Bibliometric and science mapping analysis

A bibliometric framework integrating performance analysis and science mapping techniques has been employed. Descriptive analyses have been conducted using the bibliometrix package (Aria and Cuccurullo, 2017) and its web-based interface, Biblioshiny, in the R environment. These analyses have included annual scientific production, citation patterns, the most productive authors, institutions, and countries, and source analysis based on Bradford’s Law. Collaboration patterns have been examined through co-authorship analyses at the author and country levels. For the author-level co-authorship analysis, the full counting method has been applied, with a minimum threshold of four documents per author and no minimum citation requirement. The conceptual structure of the field has been examined through keyword co-occurrence analysis using VOSviewer (van Eck and Waltman, 2010). For this analysis, the full counting method has been applied, with a minimum occurrence threshold of four. Bibliographic coupling has been conducted at the document level to identify publications sharing similar reference patterns. The full counting method has been applied, with a minimum threshold of four citations per document. Thematic evolution analysis has also been conducted to examine changes in research themes over time. Network visualizations for co-authorship, keyword co-occurrence, and bibliographic coupling analyses have been generated using VOSviewer (version 1.6.20). Association strength normalization has been applied to the VOSviewer-based network analyses.

## Results

The findings are organized into five dimensions: temporal growth, citation structure, productive contributors, geographic collaboration, and conceptual organization. This structure helps clarify how the hikikomori literature has evolved and how knowledge is organized within the field.

### Temporal growth dynamics

#### Annual publication and citation trends (2002–2025)

The temporal development of the literature was assessed by examining annual publication output together with the total citations accumulated by publications from each year.

**Figure 2:**
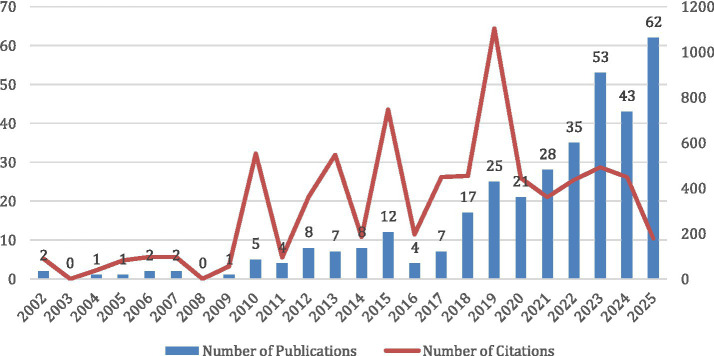
Annual distribution of publications and citations in the hikikomori literature.

Figure 2 presents annual publication output, and total citation counts by publication year. Although no lower year limit was applied during the search process, the earliest eligible publications in the final corpus were published in 2002. Publication output remained low and irregular between 2002 and 2009, with annual counts ranging from zero to three. A clearer increase was observed after 2010, and publication activity expanded markedly after 2018 despite year-to-year fluctuations. The number of publications rose from 28 in 2021 to 62 in 2025, with the highest annual output recorded in 2025 (*n* = 62). Citation counts showed a more uneven pattern. The highest total citation count was recorded for publications from 2019, with additional peaks observed for those published in 2015, 2010, and 2013. Publications from the most recent years, particularly 2024 and 2025, had accumulated fewer citations despite higher publication output. This difference is consistent with the shorter time available for recent publications to receive citations. Publication activity therefore increased substantially in the later years of the period, whereas citation impact remained uneven across publication years.

### Citation structure and influential publications

#### Most influential publications and citation distribution

The most cited publications in the final corpus were examined to identify the studies with the highest citation impact.

The ten most cited publications in the final corpus are presented in Table 1. Citation counts ranged from 138 to 305. The highest citation count was recorded for Cerniglia et al. (2017), followed by Tateno et al. (2019) and Kato et al. (2019). Several highly cited studies were directly focused on the definition, prevalence, assessment, and cross-cultural examination of hikikomori, including Teo and Gaw (2010), Koyama et al. (2010), Kato et al. (2012), and Teo et al. (2018). Publications addressing related areas, particularly internet addiction, digital behaviors, and youth social withdrawal, were also represented among the most highly cited studies. Review and measurement-oriented publications, including Li and Wong (2015) and Teo et al. (2018), were likewise included in the top ten.

**Table 1:** Top 10 most cited studies on hikikomori.

Rank	Study	Article title	Journal	Total citations
1	Cerniglia et al. (2017)	Internet Addiction in adolescence: Neurobiological, psychosocial and clinical issues	Neuroscience and Biobehavioral Reviews	305
2	Tateno et al. (2019)	Internet Addiction, Smartphone Addiction, and Hikikomori Trait in Japanese Young Adult: Social Isolation and Social Network	Frontiers in Psychiatry	235
3	Kato et al. (2019)	Hikikomori: Multidimensional understanding, assessment, and future international perspectives	Psychiatry and Clinical Neurosciences	225
4	Teo and Gaw (2010)	Hikikomori, a Japanese Culture-Bound Syndrome of Social Withdrawal? A Proposal for DSM-5	Journal of Nervous and Mental Disease	199
5	Koyama et al. (2010)	Lifetime prevalence, psychiatric comorbidity and demographic correlates of “hikikomori” in a community population in Japan	Psychiatry Research	190
6	Kato et al. (2012)	Does the ‘hikikomori’ syndrome of social withdrawal exist outside Japan? A preliminary international investigation	Social Psychiatry and Psychiatric Epidemiology	188
7	Furlong (2008)	The Japanese hikikomori phenomenon: acute social withdrawal among young people	Sociological Review	175
8	Teo et al. (2015)	Identification of the hikikomori syndrome of social withdrawal: Psychosocial features and treatment preferences in four countries	International Journal of Social Psychiatry	173
9	Teo et al. (2018)	Development and validation of the 25-item Hikikomori Questionnaire (HQ-25)	Psychiatry and Clinical Neurosciences	140
10	Li and Wong (2015)	Youth social withdrawal behavior (hikikomori): A systematic review of qualitative and quantitative studies	Australian and New Zealand Journal of Psychiatry	138

Citation counts refer to global citation metrics recorded in the merged and deduplicated Web of Science-Scopus dataset (*N* = 348). The table reports publications that remained in the final eligible corpus after topical screening. Accordingly, some highly cited publications represent broader intellectual influences connected to hikikomori, whereas others directly address the definition, prevalence, measurement, and cross-cultural assessment of hikikomori.

### Source distribution and core journals (Bradford analysis)

Bradford’s Law analysis was conducted on the final corpus of 348 publications. Before the analysis, source titles were standardized to harmonize capitalization, accent, and spelling variants across Web of Science and Scopus records. A three-zone distribution was identified across 200 publication sources. Zone 1, representing the core sources of the field, consisted of 14 journals and accounted for 117 articles. Zone 2 included 70 journals and 116 articles, whereas Zone 3 comprised 116 journals and 116 articles. A substantial proportion of the literature was concentrated within a relatively small group of journals, while the remaining publications were distributed across a much larger number of sources. This distribution was consistent with Bradford scattering, with a limited core of journals followed by progressively broader source dispersion. The journals included in the Bradford core are presented in Table 2.

**Table 2:** Core journals identified by Bradford analysis.

Rank	Journal	Articles
1	International Journal of Social Psychiatry	23
2	Frontiers in Psychiatry	23
3	Psychiatry and Clinical Neurosciences	11
4	International Journal of Environmental Research and Public Health	8
5	Current Psychology	8
6	Frontiers in Psychology	7
7	Journal of Affective Disorders	6
8	Current Opinion in Psychiatry	5
9	Asian Journal of Psychiatry	5
10	PLOS ONE	5
11	Japanese Psychological Research	4
12	Brain Sciences	4
13	Annales Médico-Psychologiques	4
14	Journal of Public Health (Germany)	4

Journals were ranked by the number of publications in the final eligible corpus (*N* = 348). Source titles were standardized before the analysis. Bradford Zone 1 comprised 14 journals and 117 articles, Zone 2 comprised 70 journals and 116 articles, and Zone 3 comprised 116 journals and 116 articles.

Table 2 presents the journals included in the Bradford core. The highest publication output was recorded for International Journal of Social Psychiatry and Frontiers in Psychiatry, with 23 articles each, followed by Psychiatry and Clinical Neurosciences with 11 articles. The remaining core sources were distributed mainly across psychiatry, psychology, public health, and related mental health fields. The concentration of 117 articles in 14 journals indicates a distinct core group of publication sources, while the broader distribution observed in Zones 2 and 3 reflects the dispersion of hikikomori research across a wide range of journals.

### Productive contributors and institutional distribution

#### Author productivity distribution

The distribution of publications across authors was examined to determine the extent to which research output was concentrated among a limited group of contributors.

Table 3 presents the empirical distribution of author productivity in the final corpus. A total of 1,070 unique authors were identified. The distribution was highly skewed, with 866 authors (80.93%) contributing a single publication and 975 authors (91.12%) contributing no more than two publications. The number of contributors declined sharply as publication counts increased. Only a small group of authors produced five or more publications, while the highest individual output reached 38 publications. This pattern was consistent with a Lotka-type productivity distribution, characterized by a broad base of occasional contributors and a much smaller group of highly productive authors. The concentration of productivity among this smaller group was examined further through individual author output and citation-based impact indicators.

**Table 3:** Empirical author productivity distribution in the hikikomori literature.

Publications per author	Authors (*n*)	% of authors	Cumulative %
1	866	80.93	80.93
2	109	10.19	91.12
3	40	3.74	94.86
4	23	2.15	97.01
5	14	1.31	98.32
6	3	0.28	98.60
7	2	0.19	98.79
8	3	0.28	99.07
9	1	0.09	99.16
10	1	0.09	99.25
11	1	0.09	99.35
14	2	0.19	99.53
15	1	0.09	99.63
17	1	0.09	99.72
19	1	0.09	99.81
27	1	0.09	99.91
38	1	0.09	100.00

A total of 1,070 unique authors were identified across the final corpus of 348 publications. Authors with a single publication accounted for 80.93% of all contributors. Author names were standardized using the abbreviated author-name field in the final merged corpus.

#### Most productive authors

Author-level publication output was examined to identify the most productive contributors to the hikikomori literature.

**Figure 3:**
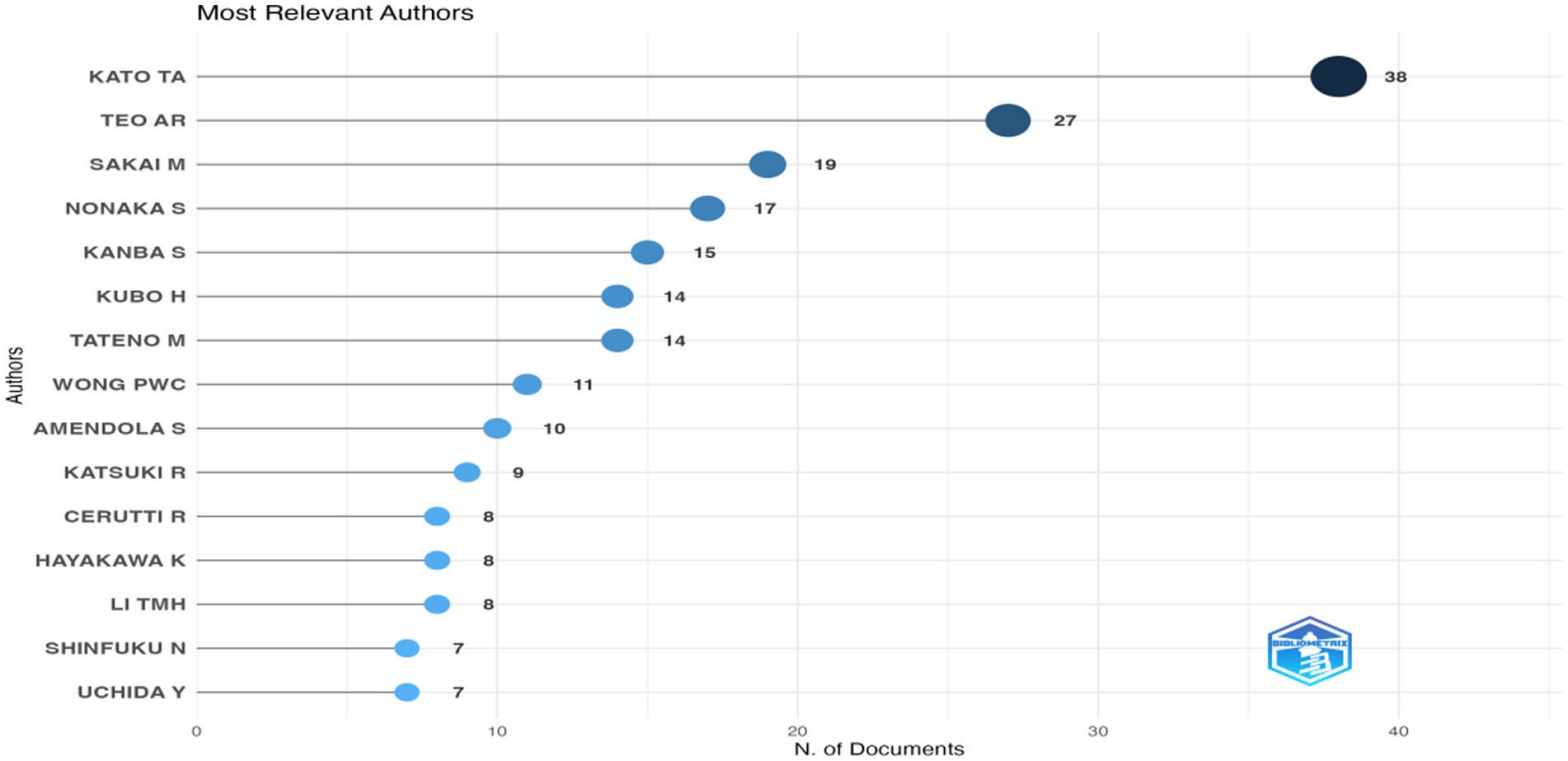
Most relevant authors in hikikomori research. The figure is based on the final merged and deduplicated corpus of 348 publications. Authors were ranked according to the number of documents.

The most productive authors in the final corpus have been presented in Figure 3. The highest publication output has been recorded for Kato TA, with 38 publications, followed by Teo AR with 27 publications and Sakai M with 19 publications. A total of 17 publications has been recorded for Nonaka S, while 15 publications have been recorded for Kanba S and 14 publications each for Kubo H and Tateno M. Wong PWC and Amendola S have also been included among the leading contributors, with 11 and 10 publications, respectively. A marked decline in publication output has been observed after the first few authors. Scientific productivity has therefore been concentrated among a relatively small group of contributors. This pattern has been consistent with the highly skewed author productivity distribution reported in Table 3, where most authors have contributed only one or two publications. At the same time, sustained publication output has been recorded for several authors, and a recognizable group of recurrent contributors has been identified within the field.

#### Author impact indicators

Citation-based impact indicators have been examined for the leading authors because publication counts alone have not been considered sufficient to capture differences in scholarly influence.

The leading authors according to productivity and citation-based impact indicators have been presented in Table 4. The highest publication output has been recorded for Kato TA, with 38 documents. The highest h-index (21) and g-index (38) values have also been recorded for this author. The highest total citation count, however, has been recorded for Teo AR, with 1,860 citations across 27 documents. A complete correspondence between publication output and citation impact has therefore not been observed. Marked differences have also been observed in citations per document. Relatively high values have been recorded for Tateno M (74.86), Kanba S (69.93), Teo AR (68.89), and Katsuki R (65.44), whereas lower values have been observed for several authors with substantial publication output. One of the highest m-index values has been recorded for Kubo H (1.222), followed by Nonaka S (1.125), Teo AR (1.059), and Amendola S (1.000). These values have reflected differences in citation impact relative to the length of each author’s publication period within the corpus. Publication productivity and citation impact have not been distributed in the same way among the leading contributors. High publication output has been concentrated among a small group of authors, whereas substantial variation has been observed in total citations, citations per document, and time-adjusted impact. A more differentiated assessment of scholarly contribution has therefore been provided by citation-based indicators than by publication counts alone.

**Table 4:** Leading authors by productivity and citation impact in the hikikomori literature.

Rank	Author	Docs	Citations	Citations per document	h-index	g-index	First year	m-index
1	Kato TA	38	1835	48.29	21	38	2012	1.400
2	Teo AR	27	1860	68.89	18	27	2010	1.059
3	Sakai M	19	354	18.63	9	18	2013	0.643
4	Nonaka S	17	214	12.59	9	14	2019	1.125
5	Kanba S	15	1,049	69.93	12	15	2012	0.800
6	Tateno M	14	1,048	74.86	12	14	2012	0.800
7	Kubo H	14	696	49.71	11	14	2018	1.222
8	Wong PWC	11	466	42.36	9	11	2015	0.750
9	Amendola S	10	114	11.40	6	10	2021	1.000
10	Katsuki R	9	589	65.44	8	9	2018	0.889
11	Li TMH	8	364	45.50	6	8	2015	0.500
12	Hayakawa K	8	318	39.75	6	8	2015	0.500
13	Cerutti R	8	99	12.38	5	8	2021	0.833
14	Shinfuku N	7	404	57.71	5	7	2012	0.333
15	Uchida Y	7	202	28.86	5	7	2011	0.312

Authors have been ranked by the number of documents in the final eligible corpus (*N* = 348). Citation counts have been based on the global citation counts available in the merged Web of Science–Scopus dataset. Citations per document have been calculated by dividing total citations by the number of documents.

#### Most productive institutions

Institutional publication output has been examined to identify the organizations that have contributed most frequently to hikikomori research.

**Figure 4:**
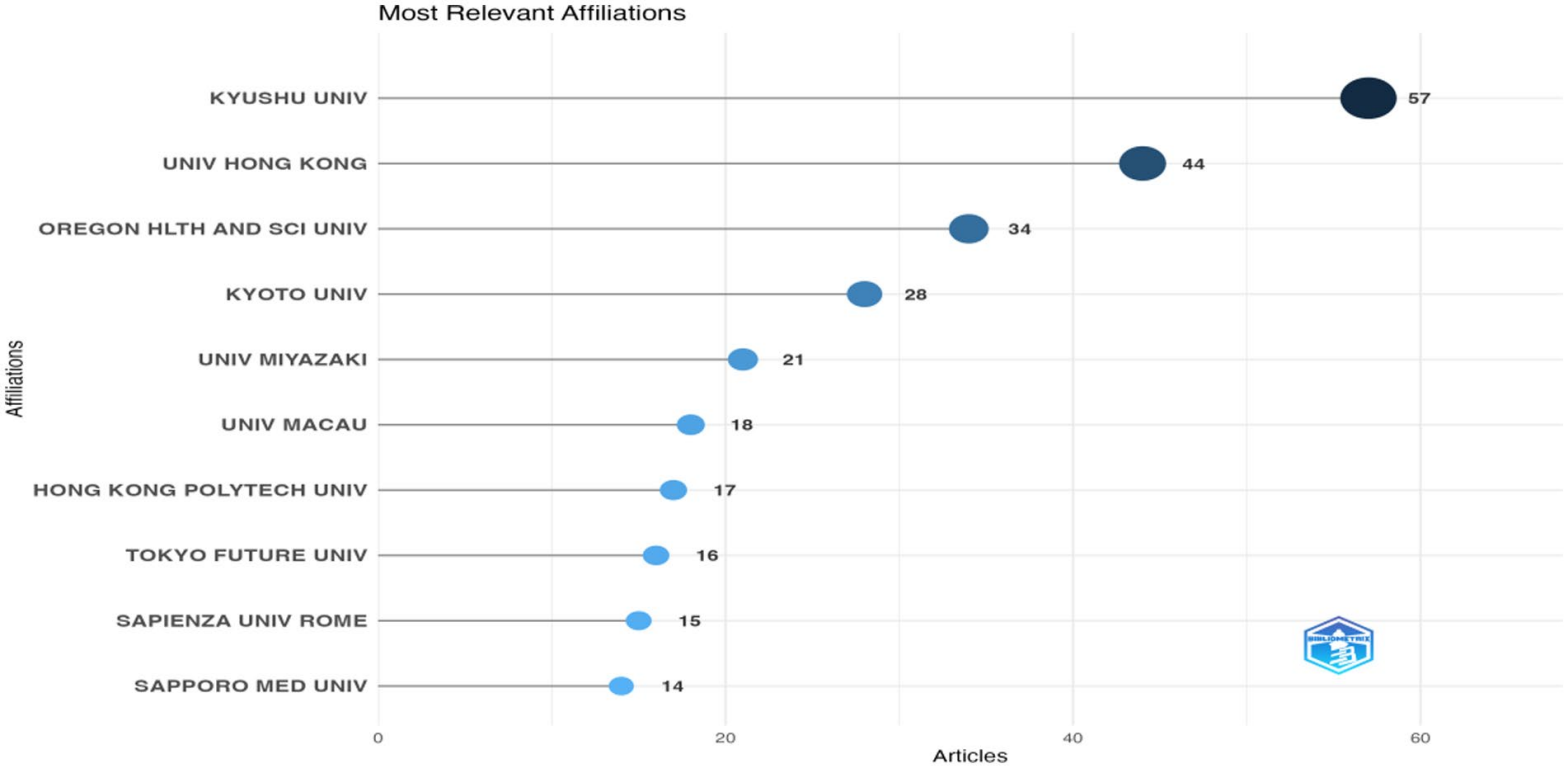
Most productive institutions in hikikomori research. The figure has been based on the final merged and deduplicated corpus of 348 publications. Institutions have been ranked according to the number of documents.

The most productive institutions in the final corpus have been presented in Figure 4. The highest publication output has been recorded for Kyushu University, with 57 publications, followed by the University of Hong Kong with 44 publications and Oregon Health and Science University with 34 publications. Kyoto University has also been identified as a major contributor, with 28 publications. Substantial publication output has further been recorded for the University of Miyazaki, University of Macau, Hong Kong Polytechnic University, Tokyo Future University, Sapienza University of Rome, and Sapporo Medical University. Institutional productivity has been concentrated among a relatively limited number of universities and health-science-oriented institutions. A particularly prominent position has been occupied by Japanese institutions, in line with the historical origins of hikikomori research. At the same time, substantial contributions have been recorded from institutions based in Hong Kong, Macau, the United States, and Italy. A broader international institutional base has therefore been evident alongside the continued prominence of Japanese universities.

### Geographic distribution and collaboration structure

#### Countries of corresponding authors

Publications have been examined according to the countries of corresponding authors to assess the geographic distribution of hikikomori research.

**Figure 5:**
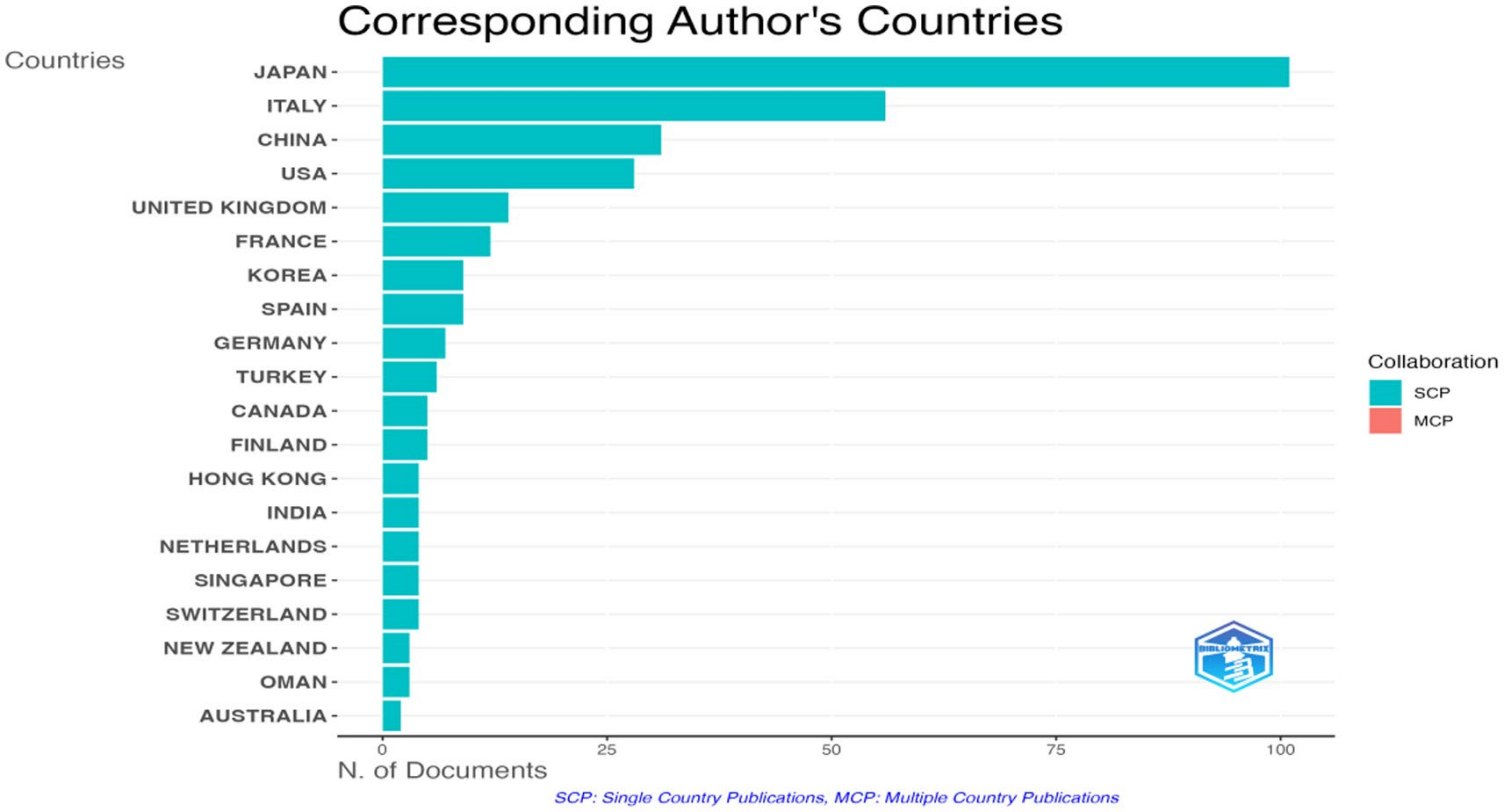
Countries of corresponding authors in hikikomori research. SCP, single-country publications; MCP, multiple-country publications. The figure has been based on the final merged and deduplicated corpus of 348 publications.

The distribution of publications by corresponding author country has been presented in Figure 5. The highest publication output has been recorded for Japan by a clear margin, followed by Italy, China, and the United States. The United Kingdom and France have also been included among the major contributing countries. Lower publication counts have been recorded for Korea, Spain, Germany, Turkey, Canada, Finland, Hong Kong, India, the Netherlands, Singapore, Switzerland, New Zealand, Oman, and Australia. A strong geographic concentration in Japan has been observed, in line with the historical origins of hikikomori research. At the same time, substantial contributions have been recorded from Italy, China, the United States, and several other European and Asian countries. A broader international distribution of research activity has therefore been evident beyond the Japanese context. Single-country publications have accounted for most of the output across the leading countries, whereas multiple-country publications have remained comparatively limited. Cross-national collaboration has therefore been less prominent than nationally based research production.

#### International collaboration network

Cross-national collaboration patterns have been mapped to examine the geographic structure of international research connections.

**Figure 6:**
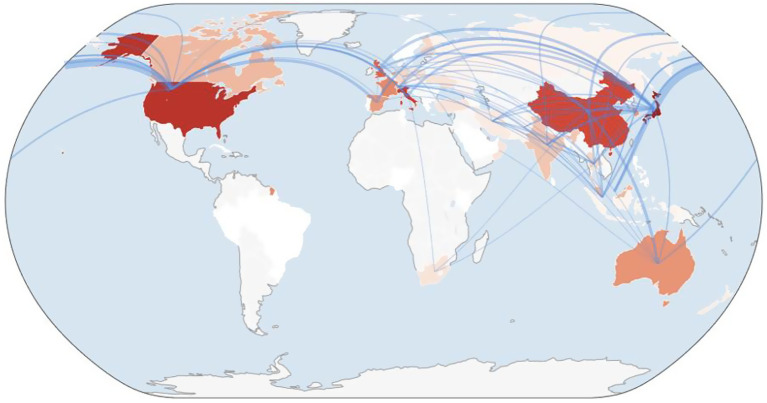
International collaboration network in hikikomori research. The figure was generated using Biblioshiny based on the final merged and deduplicated corpus of 348 publications. The map shows international collaboration links among countries represented in the corpus.

Figure 6 presents the international collaboration network in the hikikomori literature. A dense set of cross-national connections has been observed around Japan, China, the United States, and several European countries. These countries have occupied prominent positions within the network and have been linked through multiple international research connections. Collaboration links have also extended to Australia and several other parts of Asia and Europe, whereas more limited connectivity has been observed across much of Africa and South America. The collaboration structure has therefore been unevenly distributed across regions. International research connections have been concentrated among a relatively limited group of countries, while several regions have remained weakly represented in the network. Although a geographically broad pattern of participation has been identified, the strongest concentration of collaborative activity has remained within East Asia, North America, and Europe.

#### Co-authorship network of the most productive authors

Author-level collaboration has been examined to assess how the leading contributors have been connected within the co-authorship structure of the field.

**Figure 7:**
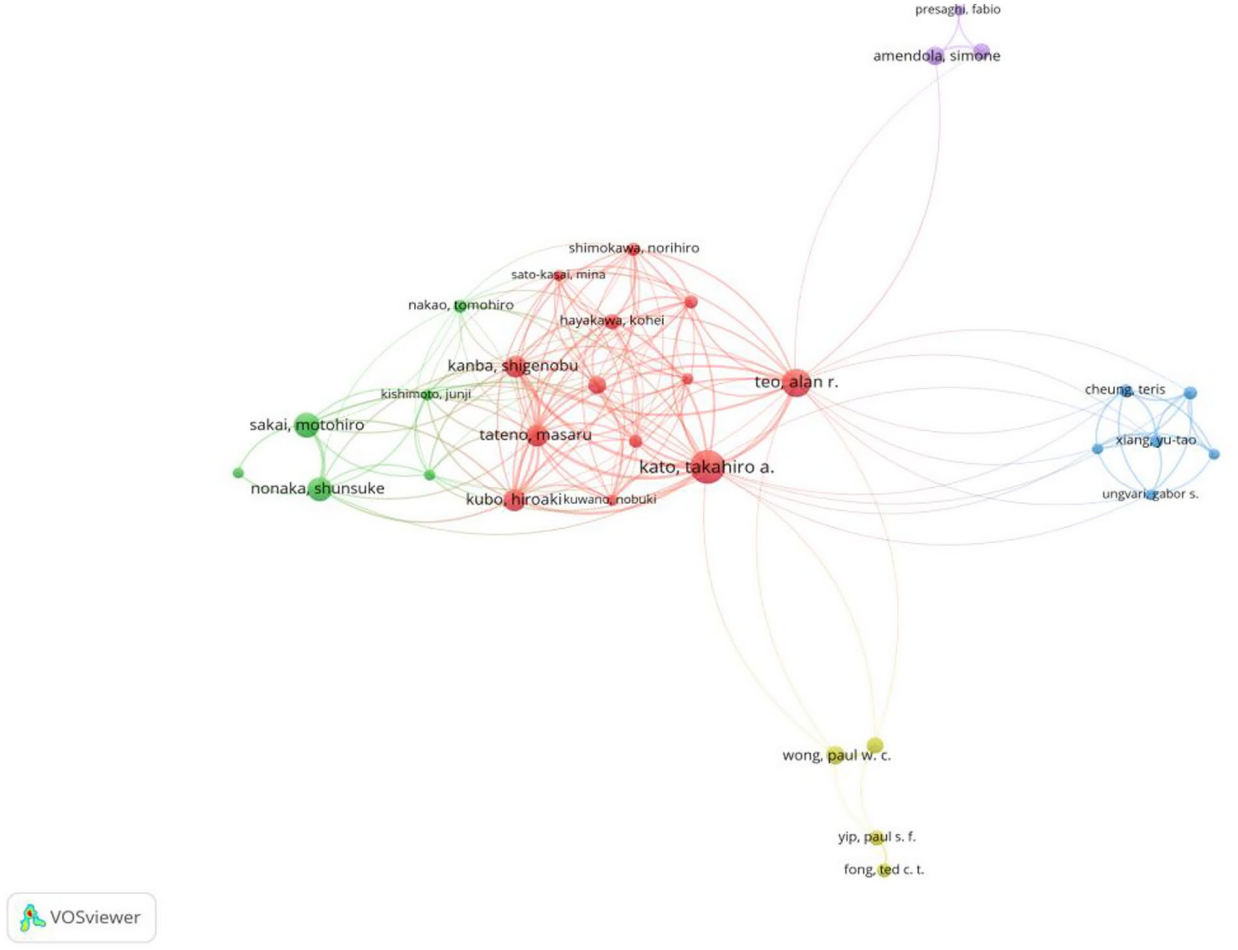
Co-authorship network of the most productive authors in the hikikomori literature. The map has been generated using VOSviewer. Authors have been used as the unit of analysis, and the full counting method has been applied. A minimum threshold of four documents per author has been set, with no minimum citation requirement. The network has included 32 authors, 5 clusters, 163 links, and a total link strength of 542. Publication volume has been represented by node size, co-authorship strength by link thickness, and author clusters by color.

The author co-authorship network has been presented in Figure 7. A dense pattern of collaboration has been observed around Kato Takahiro A., who has occupied a prominent position within the network. Strong co-authorship connections have been identified between Kato Takahiro A. and several leading contributors, including Teo Alan R., Tateno Masaru, Kanba Shigenobu, Kubo Hiroaki, and Hayakawa Kohei. A substantial share of the collaborative activity has therefore been concentrated around this closely connected group. Several distinct but interconnected subgroups have also been identified. One subgroup has included Sakai Motohiro, Nonaka Shinsuke, and related collaborators, while another has been formed around Xiang Yu-Tao, Cheung Teris, and Ungvari Gabor S. Connections between the densely linked central group and these international collaborators have been observed through Teo Alan R. A separate subgroup has included Wong Paul W. C., Yip Paul S. F., and Fong Ted C. T. Another smaller subgroup has been formed around Amendola Simone and Presaghi Fabio and has remained more peripheral to the main body of the network. The co-authorship structure has therefore been characterized by a densely connected central group together with several smaller regional and international subgroups. Collaboration has not been distributed evenly across the network, and connections between otherwise distinct groups have been concentrated around a limited number of authors. A partially centralized collaboration structure has consequently been evident within the field.

### Conceptual and intellectual structure of the field

#### Keyword co-occurrence network

The conceptual structure of the field has been examined through keyword co-occurrence mapping.

**Figure 8:**
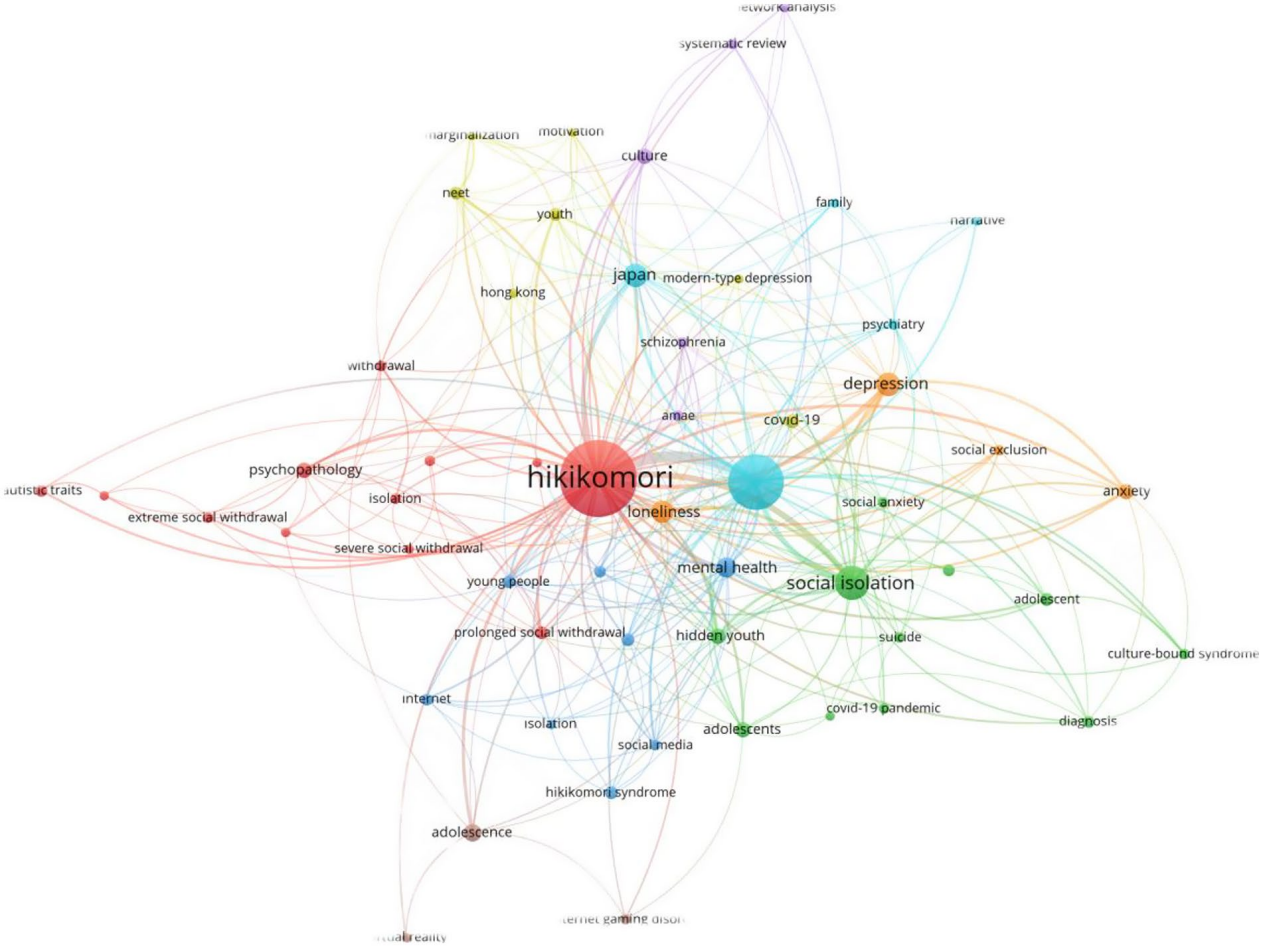
Keyword co-occurrence network in hikikomori research. The map has been generated using VOSviewer. Keywords have been used as the unit of analysis, the full counting method has been applied, and the minimum occurrence threshold has been set at four. The network has included 55 items, 8 clusters, 301 links, and a total link strength of 909. Keyword occurrence has been represented by node size, co-occurrence strength by link thickness, and clusters by color.

The keyword co-occurrence network of the hikikomori literature has been presented in Figure 8. The final network has included 55 items, 8 clusters, 301 links, and a total link strength of 909. The term *hikikomori* has occupied a central position within the network and has been strongly connected with keywords such as *social isolation*, *depression*, *Japan*, *social withdrawal*, *loneliness*, *mental health*, and *anxiety*. A dense pattern of conceptual association has therefore been observed around the core term of the field. The cluster structure has revealed several interconnected thematic domains. One cluster has been organized around social withdrawal terminology, including *social withdrawal*, *prolonged social withdrawal*, *severe social withdrawal*, *extreme social withdrawal*, *isolation*, and *psychopathology*. Another cluster has been formed around *social isolation*, *mental health*, *adolescents*, *suicide*, *diagnosis*, and *culture-bound syndrome*. A further cluster has included *depression*, *anxiety*, *loneliness*, and *social exclusion*, indicating that emotional and psychosocial dimensions have occupied a visible place in the literature.

Digital-behavior-related terms have also been represented in the network. Keywords such as *internet*, *social media*, *internet gaming disorder*, and *virtual reality* have appeared in another part of the map, where links with *hikikomori* and related psychosocial terms have also been observed. In addition, terms such as *COVID-19*, *COVID-19 pandemic*, and *hidden youth* have been included in the network, indicating that the literature has also been connected with broader public health and youth-related concerns. A multidimensional conceptual structure has therefore been evident in the keyword network. The literature has not been confined to a single diagnostic or culture-specific frame, but has instead been distributed across interrelated clinical, psychosocial, developmental, cultural, and digital-behavioral domains. The most frequent and strongly connected keywords have been presented in greater detail in Table 5.

**Table 5:** Most frequent keywords in the hikikomori literature.

Rank	Keyword	Occurrences	Total link strength	Cluster
1	Hikikomori	247	426	1
2	Social withdrawal	132	284	6
3	Social isolation	51	143	2
4	Depression	25	69	7
5	Japan	25	57	6
6	Loneliness	22	78	7
7	Mental health	17	55	3
8	Adolescence	14	23	8
9	Anxiety	11	39	7
10	Hidden youth	11	28	2
11	Psychopathology	11	24	1
12	Culture	11	21	5
13	Adolescents	10	20	2
14	COVID-19	9	18	4
15	Youth	8	29	4
16	NEET	8	22	4
17	Adolescent	8	20	2
18	Hikikomori syndrome	7	11	3
19	Prolonged social withdrawal	7	18	1
20	Internet addiction	7	18	3

Keywords have been ranked by occurrence frequency in the VOSviewer keyword co-occurrence analysis. A minimum occurrence threshold of four has been applied. The final network has included 55 items, 8 clusters, 301 links, and a total link strength of 909. Total link strength has represented the cumulative co-occurrence strength of each keyword with other keywords in the network. Minor spelling and encoding variants have been standardized before analysis.

The most frequent keywords in the hikikomori literature have been presented in Table 5. The highest occurrence and total link strength values have been recorded for *hikikomori* (247 occurrences; TLS = 426), followed by *social withdrawal* (132; TLS = 284) and *social isolation* (51; TLS = 143). These terms have formed the most prominent conceptual group within the keyword structure and have maintained extensive connections with other topics in the network. Mental health-related terms have also occupied a visible position. Relatively frequent occurrences have been recorded for *depression*, *loneliness*, *mental health*, and *anxiety*, together with substantial total link strength values. Developmental and youth-related terms, including *adolescence*, *adolescents*, *youth*, *hidden youth*, and *NEET*, have also been represented among the most frequent keywords. In addition, *culture*, *COVID-19*, *hikikomori syndrome*, *prolonged social withdrawal*, and *internet addiction* have appeared among the leading terms. The keyword distribution has therefore reflected a conceptual structure in which social withdrawal and social isolation have remained closely connected with mental health, developmental, cultural, public health, and digital-behavioral themes.

#### Bibliographic coupling network

Bibliographic coupling has been used to examine relationships among documents based on shared reference patterns.

**Figure 9:**
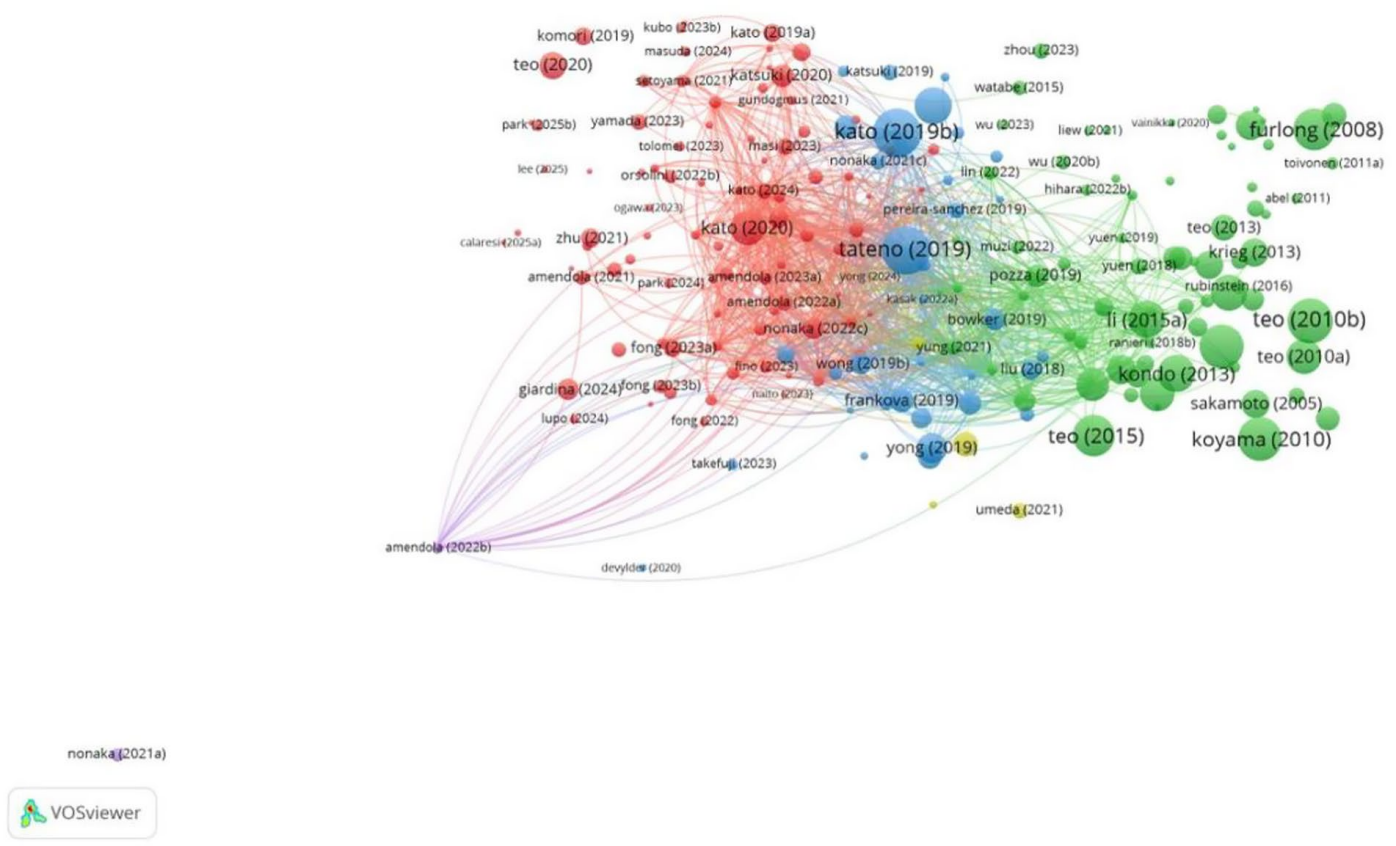
Bibliographic coupling network of documents in hikikomori research. The map has been generated using VOSviewer. Documents have been used as the unit of analysis, and the full counting method has been applied. A minimum threshold of four citations per document has been set. The network has included 204 documents, 5 clusters, 16,048 links, and a total link strength of 73,626. Document weight has been represented by node size, coupling strength by link thickness, and average publication year by overlay color.

The document-level bibliographic coupling network has been presented in Figure 9. The network has included 204 documents, 5 clusters, 16,048 links, and a total link strength of 73,626. Documents have been connected when shared references have been identified, with stronger coupling representing greater overlap in cited literature. A densely interconnected coupling structure has therefore been observed across the network. Several prominent nodes have been identified across the network. In the VOSviewer map, these nodes have been labeled as Tateno et al. (2019), Kato et al. (2019), Kato et al. (2020), Kondo (2013), Li and Wong (2015), Teo (2010), Teo (2013), Teo et al. (2015), Koyama et al. (2010), and Furlong (2008). Tateno et al. (2019) and Kato et al. (2019) have been positioned within densely connected areas near the center of the network, while Kondo (2013), Li and Wong (2015), several Teo-labeled documents, Koyama et al. (2010), and Furlong (2008) have been located within the large cluster on the right side of the map. Other highly connected nodes, including documents labeled Bowker (2019), Pozza et al. (2019), and Wong (2019), have been positioned between major parts of the network. The five-cluster structure has shown that the literature has been organized into several distinct but strongly interconnected document groupings. Extensive coupling links have remained visible across the clusters, particularly around publications located in the central part of the network. A substantial degree of overlap in shared reference bases has therefore been evident, alongside differentiation among connected lines of research.

#### Thematic evolution

**Figure 10:**
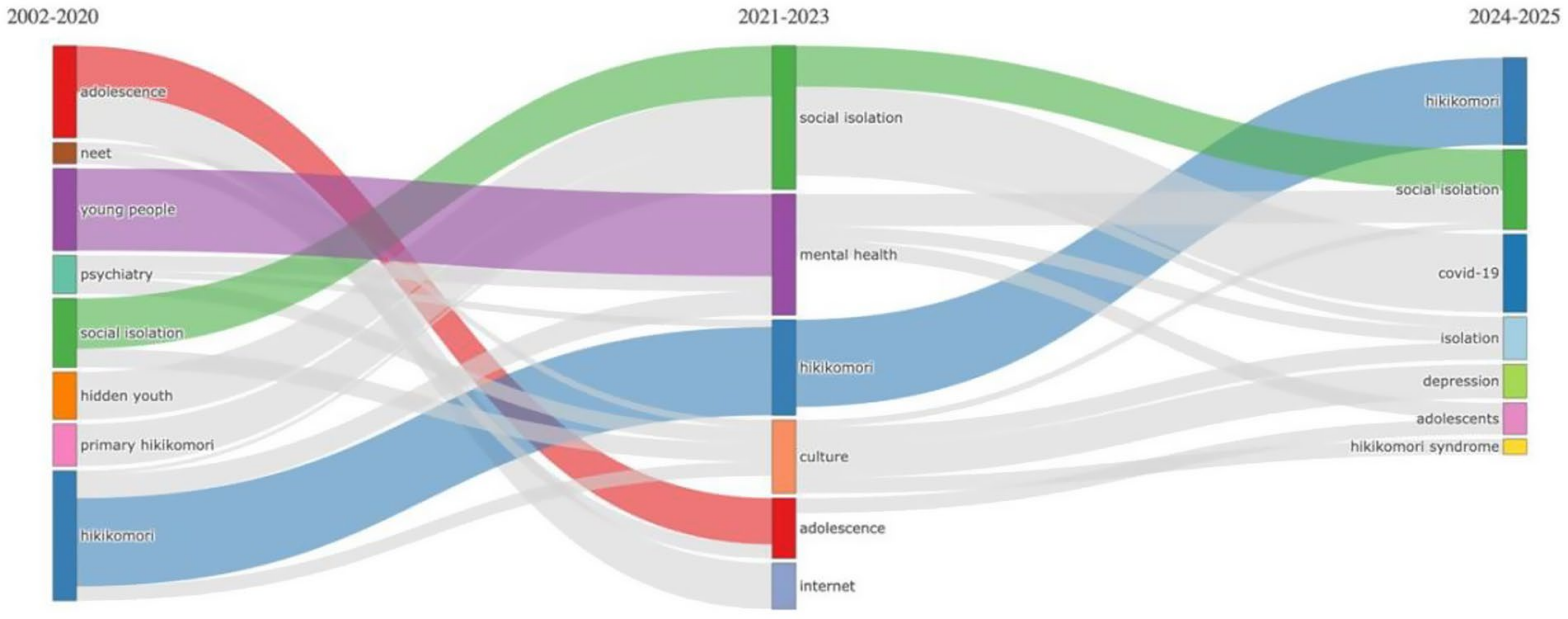
Thematic evolution of hikikomori research across time periods. The thematic evolution map has been generated using Biblioshiny based on author keywords. Generic indexing terms, methodological labels, measurement-related terms, and broad geographic descriptors have been removed before analysis. The flows have represented thematic continuity and transformation across the periods, and wider flows have indicated stronger thematic connections.

The thematic evolution of hikikomori research across three time periods has been presented in Figure 10. The analysis has covered the periods 2002–2020, 2021–2023, and 2024–2025. In the first period (2002–2020), the main themes have been organized around *hikikomori*, *social isolation*, *adolescence*, *young people*, *NEET*, *hidden youth*, *primary hikikomori*, and *psychiatry*. In this period, the literature has been structured mainly around youth-related social withdrawal, marginalization, psychiatric interpretation, and distinctions between primary hikikomori and related forms of withdrawal. In the second period (2021–2023), *hikikomori* has remained a central theme, while a more differentiated thematic structure has been observed. *Social isolation* and *mental health* have occupied prominent positions, and *culture*, *adolescence*, and *internet* have also been represented. Continuity in the conceptual core has therefore been maintained, while a broader psychosocial and clinical orientation has also become more visible.

In the most recent period (2024–2025), the thematic structure has been organized around *hikikomori*, *social isolation*, *COVID-19*, *isolation*, *depression*, *adolescents*, and *hikikomori syndrome*. Greater attention has therefore been given to pandemic-related conditions, depressive symptoms, adolescent populations, and syndrome-oriented conceptualization. The thematic flows have also shown that earlier themes centered on *hikikomori* and *social isolation* have remained connected with these more recent topics. Across the three periods, both continuity and diversification have been evident. *Hikikomori* and *social isolation* have remained the most persistent themes, while *mental health*, *COVID-19*, *depression*, and *adolescents* have become more visible in the later periods. The thematic evolution pattern has therefore shown that the literature has been extended from an earlier concentration on youth withdrawal and psychiatric framing toward a broader configuration that has included mental health, developmental, and public health concerns.

## Discussion

This study has provided a systematic examination of the growth dynamics, productivity patterns, geographic distribution, collaboration structure, and conceptual and intellectual development of the hikikomori literature. The findings have shown that publication output has remained low and irregular during the early years of the field, has become more visible after 2010, and has expanded markedly after 2018. The highest annual publication output has been recorded in 2025, with 62 publications. This growth has been associated with a gradual shift in the conceptualization of hikikomori within the literature. Earlier research has largely been rooted in the Japanese context, and hikikomori has often been examined as a culture-specific form of severe social withdrawal (Teo, 2010; Teo and Gaw, 2010). As broader diagnostic, psychosocial, and cross-cultural perspectives have been developed, greater scholarly attention has been directed toward the phenomenon. Evidence from different national contexts has also challenged a strictly culture-bound interpretation, and hikikomori has increasingly been examined as a cross-cultural and global mental health concern (Dong et al., 2022; Kato et al., 2019).

The acceleration in publication output has been evident since before the COVID-19 pandemic but may have been further reinforced by growing attention to social isolation and prolonged withdrawal during and after the pandemic period. Pandemic-related restrictions and reduced in-person contact have intensified concerns about withdrawal-related behaviors and their possible mental health consequences (Herold et al., 2021; Ogawa et al., 2023). Existing conceptual frameworks have also been reconsidered in light of emerging distinctions between pathological and non-pathological forms of hikikomori (Kato et al., 2024). In addition, the ranking of highly cited publications has highlighted the influence of earlier studies that shaped key conceptual debates in the field. Teo and Gaw (2010) systematically examined hikikomori within the culture-bound syndrome debate, whereas Kato et al. (2012) extended the discussion by examining its relevance across multiple national and cultural contexts.

The analysis of the most cited publications has shown that the hikikomori literature has been connected not only with studies focused directly on the phenomenon but also with adjacent areas such as internet addiction, digital behaviors, youth social withdrawal, and measurement. The technological dimension has been particularly visible in highly cited work in which internet and smartphone use have been linked with hikikomori tendencies (Tateno et al., 2019). This association has also been examined in more recent studies based on contemporary samples (Lin et al., 2025; Gavin et al., 2025). Source distribution has revealed a relatively concentrated publication structure. Bradford’s Law analysis has identified 14 core journals accounting for 117 of the 348 publications, with *International Journal of Social Psychiatry* and *Frontiers in Psychiatry* occupying the leading positions. At the same time, publications have been widely dispersed across the second and third Bradford zones. Hikikomori research has therefore been represented across psychiatric, psychological, public health, and related interdisciplinary outlets. This pattern may reflect the strong clinical and psychiatric orientation of hikikomori research. Studies in the field have examined diagnostic boundaries, psychiatric comorbidity, psychopathology, and functional impairment, while also addressing developmental, psychosocial, and societal dimensions of severe social withdrawal (Kato et al., 2024; Muris and Ollendick, 2023; Tolomei et al., 2023). This breadth of research may help explain why publications are concentrated in psychiatric journals while also appearing across psychological, public health, and interdisciplinary outlets.

Author productivity has also been highly uneven. A total of 80.93% of authors have contributed only one publication, whereas sustained output has been concentrated among a much smaller group of contributors. This pattern has been consistent with a Lotka-type productivity distribution. Among the leading authors, Kato TA has combined the highest publication output with the highest h-index and g-index values, while Teo AR has recorded the highest total citation count despite a lower number of publications. Publication productivity and citation impact have therefore not been distributed in the same way across the leading contributors. The prominence of Kato TA may partly reflect the central role of Japan in the emergence and institutional development of hikikomori research, including sustained clinical and research activity at Kyushu University (Kato et al., 2024). Teo AR’s high citation impact, despite a lower publication count, may instead be related to the influence of earlier conceptual and diagnostic work that helped place hikikomori within broader international and cross-cultural debates (Teo and Gaw, 2010).

Institutional productivity has also been concentrated among a relatively limited number of universities. The highest publication output has been recorded for Kyushu University, followed by the University of Hong Kong and Oregon Health and Science University. This pattern has been broadly consistent with earlier bibliometric evidence, which likewise identified Kyushu University as the leading institutional contributor and placed Oregon Health and Science University and the University of Hong Kong among the most productive centers in the field (Cai et al., 2023). The prominence of Kyushu University may also be understood in relation to the historical and institutional development of hikikomori research in Japan, including the establishment of a dedicated hikikomori research clinic at Kyushu University Hospital (Kato et al., 2024). A broader international institutional base has also been evident through substantial contributions from institutions in Hong Kong, the United States, Macau, and Italy. The findings have therefore shown that the institutional base of the field has remained strongly anchored in Japan while also involving a wider range of international research settings.

Findings on geographic distribution have shown that Japan has remained the dominant contributor, while substantial publication activity has also been recorded in Italy, China, the United States, and several other European and Asian countries. The strong presence of Italy has been consistent with the growing body of European research on hikikomori documented in clinical and psychosocial studies (Benarous et al., 2022; Santona et al., 2023; Tolomei et al., 2023). Contributions from China have likewise demonstrated that the research base has not been confined to the Japanese context (Hu et al., 2022). At the same time, single-country publications have accounted for most of the output, while multi-country collaboration has remained comparatively limited. Research production has therefore continued to be organized largely within national settings despite a broader international distribution. The co-authorship network has provided further insight into this pattern. A dense concentration of collaborative activity has been observed around Kato TA and closely connected contributors. Teo AR has been linked with both the Japanese-centered group and several international collaborators, while the Italian subgroup has remained more peripheral to the main body of the network. These patterns have shown that international collaboration has been developed unevenly, with connections across regional groups concentrated around a limited number of highly connected authors.

Keyword analysis has also revealed the prominence of terms such as *depression anxiety loneliness* and digital-behavior-related concepts. Hikikomori has therefore been examined alongside psychiatric and psychosocial dimensions rather than solely through the lens of social withdrawal. This interpretation has been supported by clinical evidence in which depression anxiety and other co-occurring conditions have frequently been identified (Nonaka et al., 2025; Tolomei et al., 2023; Yasuma et al., 2021). Associations between loneliness and suicidal ideation have also been documented in different samples (Fong and Yip, 2023). Finally the bibliographic coupling network has revealed substantial overlap in the reference bases used across the literature. A shared intellectual foundation has therefore been evident lthough several connected lines of research have continued to coexist within the field.

Thematic evolution analysis has shown that hikikomori and social isolation have remained persistent themes across the examined periods. In the earliest period, themes were organized mainly around youth-related withdrawal, social marginalization, psychiatric interpretation, and concepts such as NEET, hidden youth, and primary hikikomori. In later periods, themes related to mental health, COVID-19, depression, and adolescents became more prominent. This pattern suggests a broadening of the research agenda from earlier debates about cultural specificity and youth withdrawal toward a wider set of clinical, psychosocial, developmental, and public health concerns (Teo and Gaw, 2010; Kato et al., 2024; Muris and Ollendick, 2023; Pupi et al., 2025). Efforts toward greater conceptual and clinical standardization have also become more visible in recent years. The Hikikomori Diagnostic Evaluation (HiDE) has been proposed as a structured, clinician-administered approach to assessing pathological social withdrawal (Teo et al., 2023), while systematic and meta-analytic work has synthesized evidence on functioning, mental health symptoms, and interpersonal interactions associated with hikikomori (Nonaka et al., 2025). Recent work has also examined hikikomori through transcultural and digital perspectives, including the changing role of online interaction, gaming, and post-pandemic social life (Nagai et al., 2024).

## Limitations

Several limitations should be considered when the findings are interpreted. The analysis has been limited to publications indexed in Web of Science and Scopus. Although these databases have provided broad coverage, relevant studies indexed only in regional or specialized databases may not have been captured. Only English-language publications have also been included. Given the substantial body of hikikomori research produced in Japan and other non-English-speaking contexts, studies published in local languages may have been underrepresented, together with region-specific perspectives that might not be fully visible in the international literature. Because final eligibility required an explicit reference to “hikikomori,” conceptually related studies using only broader social withdrawal terminology may also have been underrepresented. The geographic and collaboration patterns should therefore be interpreted as reflecting explicitly identified hikikomori research rather than the entire literature on severe social withdrawal. Citation-based indicators have been influenced by publication timing, as more recent studies have had less opportunity to accumulate citations. Differences in coverage and citation indexing between Web of Science and Scopus may also have affected citation-based measures. In addition, the analysis has depended on the quality and consistency of the metadata provided by the two databases. Although author names, affiliations, source titles, and keywords have been cleaned and standardized, some residual inconsistencies may have remained. Certain analytical decisions should also be taken into account. The standardization of keywords has required judgments about whether related terms should be merged or retained separately, and these decisions may have influenced the resulting conceptual structure. Likewise, minimum occurrence thresholds and network inclusion criteria may have affected which items were represented in the maps. Despite these limitations, a broader view of the field has been provided through the combined use of two major databases and several complementary bibliometric approaches.

## Conclusion

This study has provided a comprehensive bibliometric overview of the hikikomori literature and has shown that the field has expanded markedly over the past decade. Scientific production has been concentrated among a relatively limited group of authors, institutions, and countries, while international collaboration has been unevenly distributed. At the same time, the conceptual structure of the literature has broadened to include mental health, psychosocial functioning, social isolation, youth-related concerns, and digital behaviors. Hikikomori has increasingly been examined beyond a culture-specific framework. A broader and more multidimensional research perspective has emerged, with clinical, psychosocial, developmental, cultural, and digital dimensions becoming more visible across the literature. This development has highlighted the need for greater cross-cultural and interdisciplinary engagement in future research. The relatively limited level of multi-country collaboration has also pointed to the importance of stronger connections among regional research groups, particularly where cross-national links remain limited. Several priorities have been identified for future research. Broader inclusion of non-English publications and the use of additional regional or specialized databases may provide a more balanced representation of the literature. Comparative and cross-cultural studies are also needed to clarify how hikikomori has been conceptualized and experienced across different social and cultural contexts. Greater attention should be given to the relationship between hikikomori and digital environments, while longitudinal research may help clarify developmental pathways and changes over time.

## Data Availability

The data supporting the conclusions of this article will be made available by the author, without undue reservation, in the form of processed and aggregated datasets. Requests to access these datasets should be directed to fatihalpcebeci@gmail.com.

## References

[ref1] Aria M. Cuccurullo C. (2017). Bibliometrix: an R-tool for comprehensive science mapping analysis. J. Informetr. 11, 959–975. doi: 10.1016/j.joi.2017.08.007

[ref2] Arslankoç S. Karakaya Altıok Ş. Cebeci F. Meydan S. Bolat G. B. Arıcı A. . (2026). A psychosocial model of hikikomori: mediating and moderating roles of psychological resilience and sociodemographic factors among Turkish adults. J. Health Psychol.:13591053261452614. doi: 10.1177/13591053261452614, 42224448

[ref3] Benarous X. Guedj M.-J. Cravero C. Jakubowicz B. Brunelle J. Suzuki K. . (2022). Examining the hikikomori syndrome in a French sample of hospitalized adolescents with severe social withdrawal and school refusal behavior. Transcult. Psychiatry 59, 831–843. doi: 10.1177/13634615221111633, 35866212

[ref4] Cai H. Sha S. Zhang Q. Si T. L. Liu Y.-F. Zheng W.-Y. . (2023). Hikikomori: a perspective from bibliometric analysis. Psychiatry Clin. Neurosci. 77, 541–549. doi: 10.1111/pcn.13573, 37350640

[ref5] Cebeci F. Arıcı A. Meydan S. Karakaya Altıok Ş. Arslankoç S. Lotfi S. . (2026). The mediating role of learned meaninglessness in the relationship between hikikomori and unemployment anxiety among Turkish university students. Int. J. Educ. Vocat. Guid., 1–26. doi: 10.1007/s10775-026-09791-5, 30311153

[ref6] Cerniglia L. Zoratto F. Cimino S. Laviola G. Ammaniti M. Adriani W. (2017). Internet addiction in adolescence: neurobiological, psychosocial and clinical issues. Neurosci. Biobehav. Rev. 76, 174–184. doi: 10.1016/j.neubiorev.2016.12.024, 28027952

[ref7] Dong B. Li D. Baker G. B. (2022). Hikikomori: a society-bound syndrome of severe social withdrawal. Psychiatry Clinic. Psychopharmacol. 32, 167–173. doi: 10.5152/pcp.2022.22429, 38764869 PMC11099621

[ref8] Donthu N. Kumar S. Mukherjee D. Pandey N. Lim W. M. (2021). How to conduct a bibliometric analysis: an overview and guidelines. J. Bus. Res. 133, 285–296. doi: 10.1016/j.jbusres.2021.04.070

[ref9] Du Q. Zhao R. Wan Q. Li S. Li H. Wang D. . (2024). Protocol for conducting bibliometric analysis in biomedicine and related research using CiteSpace and VOSviewer software. STAR Protoc. 5:103269. doi: 10.1016/j.xpro.2024.103269, 39240711 PMC11408372

[ref10] Fong T. C. T. Yip P. S. F. (2023). Prevalence of hikikomori and associations with suicidal ideation, suicide stigma, and help-seeking among 2,022 young adults in Hong Kong. Int. J. Soc. Psychiatry 69, 1768–1780. doi: 10.1177/00207640231174376, 37191282

[ref11] Furlong A. (2008). The Japanese hikikomori phenomenon: acute social withdrawal among young people. Sociol. Rev. 56, 309–325. doi: 10.1111/j.1467-954X.2008.00790.x

[ref12] Gavin J. Brosnan M. Joiner R. (2025). Pathological and non-pathological hikikomori: social media use, digital engagement, and therapeutic implications. Front. Psych. 16:1596504. doi: 10.3389/fpsyt.2025.1596504, 40852141 PMC12367765

[ref13] Herold M. Herold R. Csuta C. Tényi T. (2021). Hikikomori: a possible mental health consequence of the COVID-19 epidemic. Orv. Hetil. 162, 1637–1642. doi: 10.1556/650.2021.32357, 34633984

[ref14] Hihara S. Kambara K. Umemura T. Handa K. Sugimura K. (2022). Diverse trajectories of hikikomori symptoms during job search and the role of identity distress: three wave longitudinal research. Front. Psych. 13:897806. doi: 10.3389/fpsyt.2022.897806, 35873266 PMC9301010

[ref15] Hu X. Fan D. F. Shao Y. (2022). Social withdrawal (hikikomori) conditions in China: a cross-sectional online survey. Front. Psychol. 13:826945. doi: 10.3389/fpsyg.2022.826945, 35360625 PMC8963962

[ref16] Hussain W. M. H. W. (2023). Evolution and trends of hikikomori: a bibliometrics analysis. Int. J. Psychiatry Clin. Pract. 27, 385–396. doi: 10.1080/13651501.2023.2233580, 37452649

[ref17] Kasak M. Hacıosmanoğlu C. D. Tural Hesapcioglu S. Ceylan M. F. (2022). Loneliness in modern world: a case study of hikikomori from Turkey. Turk. J. Clin. Psychol. 25, 117–122. doi: 10.5505/kpd.2022.59265

[ref18] Kato T. A. Kanba S. Teo A. R. (2019). Hikikomori: multidimensional understanding, assessment and future international perspectives. Psychiatry Clin. Neurosci. 73, 427–440. doi: 10.1111/PCN.12895, 31148350

[ref19] Kato T. A. Kanba S. Teo A. R. (2020). Defining pathological social withdrawal: proposed diagnostic criteria for hikikomori. World Psychiatry 19, 116–117. doi: 10.1002/WPS.20705, 31922682 PMC6953582

[ref20] Kato T. A. Sartorius N. Shinfuku N. (2024). Shifting the paradigm of social withdrawal: a new era of coexisting pathological and non-pathological hikikomori. Curr. Opin. Psychiatry 37, 177–184. doi: 10.1097/yco.0000000000000929, 38415743 PMC10990035

[ref21] Kato T. A. Tateno M. Shinfuku N. Fujisawa D. Teo A. R. Sartorius N. . (2012). Does the 'hikikomori' syndrome of social withdrawal exist outside Japan? A preliminary international investigation. Soc. Psychiatry Psychiatr. Epidemiol. 47, 1061–1075. doi: 10.1007/S00127-011-0411-7, 21706238 PMC4909153

[ref22] Koyama A. Miyake Y. Kawakami N. Tsuchiya M. Tachimori H. Takeshima T. . (2010). Lifetime prevalence, psychiatric comorbidity and demographic correlates of "hikikomori" in a community population in Japan. Psychiatry Res. 176, 69–74. doi: 10.1016/j.psychres.2008.10.019, 20074814

[ref23] Li T. M. Wong P. W. (2015). Youth social withdrawal behavior (hikikomori): a systematic review of qualitative and quantitative studies. Aust. N. Z. J. Psychiatry 49, 595–609. doi: 10.1177/0004867415581179, 25861794

[ref24] Lin P. K. F. Lim P. K. H. Yow Y. J. Lee J. Akiyama Y. A. (2025). From pixels to isolation: exploring the relationship between technological addiction, depression, hikikomori risk factors, and hikikomori tendencies among young adults. Int. J. Soc. Psychiatry 71, 1598–1609. doi: 10.1177/00207640251347470, 40556522 PMC12634892

[ref25] Malagón-Amor Á. Martín-López L. M. Córcoles D. González A. Bellsolà M. Teo A. R. . (2018). A 12-month study of the hikikomori syndrome of social withdrawal: clinical characterization and different subtypes proposal. Psychiatry Res. 270, 1039–1046. doi: 10.1016/j.psychres.2018.03.060, 29615267

[ref26] Moral-Muñoz J. A. Herrera-Viedma E. Santisteban-Espejo A. Cobo M. J. (2020). Software tools for conducting bibliometric analysis in science: an up-to-date review. El Prof. Inf. 29:e290103. doi: 10.3145/epi.2020.ene.03

[ref27] Muris P. Ollendick T. H. (2023). Contemporary hermits: a developmental psychopathology account of extreme social withdrawal (hikikomori) in young people. Clin. Child. Fam. Psychol. Rev. 26, 459–481. doi: 10.1007/s10567-023-00425-8, 36653555 PMC9848719

[ref28] Nagai Y. Kartar A. Pfaff M. Elkholy H. (2024). The paradox of hikikomori through a transcultural lens. BJPsych Int. 22, 22–24. doi: 10.1192/bji.2024.38, 40290477 PMC12022855

[ref29] Neoh M. J. Y. Carollo A. Lim M. Esposito G. (2023). Hikikomori: a scientometric review of 20 years of research. Int. J. Environ. Res. Public Health 20:5657. doi: 10.3390/ijerph20095657, 37174175 PMC10177810

[ref30] Nonaka S. Kubo H. Takeda T. Sakai M. (2025). Functioning, disability, and health of individuals with hikikomori (prolonged social withdrawal) and their families: a systematic review and meta-analysis of case-control studies. Int. J. Soc. Psychiatry 71, 622–641. doi: 10.1177/00207640241310189, 39840577 PMC12171063

[ref31] Nonaka S. Takeda T. Sakai M. (2022). Who are hikikomori? Demographic and clinical features of hikikomori (prolonged social withdrawal): a systematic review. Aust. N. Z. J. Psychiatry 56, 1542–1554. doi: 10.1177/00048674221085917, 35332798

[ref32] Ogawa T. Shiratori Y. Midorikawa H. Aiba M. Sugawara D. Kawakami N. . (2023). A survey of changes in the psychological state of individuals with social withdrawal (hikikomori) in the context of the COVID pandemic. COVID 3, 1161–1172. doi: 10.3390/covid3080082

[ref33] Orsolini L. Longo G. Bellagamba S. Kato T. A. Volpe U. (2023). Hikikomori-like social withdrawal: an Italian case report. Psychiatry Clin. Neurosci. 77, 510–510. doi: 10.1111/pcn.13570, 37254899

[ref9001] Page M. J. McKenzie J. E. Bossuyt P. M. Boutron I. Hoffmann T. C. Mulrow N. . (2021). The PRISMA 2020 statement: An updated guideline for reporting systematic reviews. BMJ 372:71. doi: 10.1136/bmj.n71 PMC8005924 33782057

[ref34] Pozza A. Coluccia A. Kato T. A. Gaetani M. Ferretti F. (2019). The 'hikikomori' syndrome: worldwide prevalence and co-occurring major psychiatric disorders: a systematic review and meta-analysis protocol. BMJ Open 9:e025213. doi: 10.1136/BMJOPEN-2018-025213, 31542731 PMC6756420

[ref35] Pupi V. Bressi C. Porcelli P. Rossetti M. G. Bellani M. Trabacca A. . (2025). Hikikomori (prolonged social withdrawal) and co-occurring psychiatric disorders and symptoms in adolescents and young adults: a scoping review. Compr. Psychiatry 138:152573. doi: 10.1016/j.comppsych.2024.152573 39823783

[ref36] Saito T. (1998). *Shakaiteki Hikikomori: Owaranai Shishunki* [Social Withdrawal: Adolescence without End]. Tokyo: PHP Kenkyūjo.

[ref37] Sangadji S. S. (2023). Foundations of bibliometric writing: a comprehensive guide for novice researchers. SCIENTIA J. Multi Discip. Sci. 2, 95–107. doi: 10.62394/scientia.v2i2.79

[ref38] Santona A. Lionetti F. Tognasso G. Fusco C. Gorla L. (2023). Sensitivity and attachment in an Italian sample of hikikomori adolescents and young adults. Int. J. Environ. Res. Public Health 20:6148. doi: 10.3390/ijerph20126148, 37372736 PMC10298558

[ref39] Tateno M. Teo A. R. Ukai W. Kanazawa J. Katsuki R. Kubo H. . (2019). Internet addiction, smartphone addiction, and hikikomori trait in Japanese young adult: social isolation and social network. Front. Psych. 10:455. doi: 10.3389/FPSYT.2019.00455, 31354537 PMC6635695

[ref40] Teo A. R. (2010). A new form of social withdrawal in Japan: a review of hikikomori. Int. J. Soc. Psychiatry 56, 178–185. doi: 10.1177/0020764008100629, 19567455 PMC4886853

[ref41] Teo A. R. Chen J. I. Kubo H. Katsuki R. Sato-Kasai M. Shimokawa N. . (2018). Development and validation of the 25-item hikikomori questionnaire (HQ-25). Psychiatry Clin. Neurosci. 72, 780–788. doi: 10.1111/pcn.12691, 29926525 PMC6221010

[ref42] Teo A. R. Fetters M. D. Stufflebam K. Tateno M. Balhara Y. P. S. Choi T. Y. . (2015). Identification of the hikikomori syndrome of social withdrawal: psychosocial features and treatment preferences in four countries. Int. J. Soc. Psychiatry 61, 64–72. doi: 10.1177/0020764014535758, 24869848 PMC5573567

[ref43] Teo A. R. Gaw A. C. (2010). Hikikomori, a Japanese culture-bound syndrome of social withdrawal? A proposal for DSM-5. J. Nerv. Ment. Dis. 198, 444–449. doi: 10.1097/NMD.0B013E3181E086B1, 20531124 PMC4912003

[ref44] Teo A. R. Horie K. Kurahara K. Kato T. A. (2023). The hikikomori diagnostic evaluation (HiDE): a proposal for a structured assessment of pathological social withdrawal. World Psychiatry 22, 478–479. doi: 10.1002/wps.21123, 37713577 PMC10503919

[ref45] Tolomei G. Masi G. Milone A. Fantozzi P. Viglione V. Narzisi A. . (2023). Hikikomori (severe social withdrawal) in Italian adolescents: clinical features and follow-up. Children 10:1669. doi: 10.3390/children10101669, 37892332 PMC10605260

[ref46] van Eck N. J. Waltman L. (2010). Software survey: VOSviewer, a computer program for bibliometric mapping. Scientometrics 84, 523–538. doi: 10.1007/s11192-009-0146-3, 20585380 PMC2883932

[ref47] Yasuma N. Watanabe K. Nishi D. Ishikawa H. Tachimori H. Takeshima T. . (2021). Psychotic experiences and hikikomori in a nationally representative sample of adult community residents in Japan: a cross-sectional study. Front. Psych. 11:602678. doi: 10.3389/FPSYT.2020.602678 PMC7878546 33584370

[ref48] Zupic I. Čater T. (2015). Bibliometric methods in management and organization. Organ. Res. Methods 18, 429–472. doi: 10.1177/1094428114562629

